# Wetting Behavior Driven by Surface Morphology Changes Induced by Picosecond Laser Texturing

**DOI:** 10.3390/ma17081719

**Published:** 2024-04-09

**Authors:** Carmelo Corsaro, Gabriele Orlando, Gabriele Costa, Mariangela Latino, Francesco Barreca, Angela Maria Mezzasalma, Fortunato Neri, Enza Fazio

**Affiliations:** 1 Department of Mathematical and Computational Sciences, Physics Science and Earth Science, University of Messina, Viale F. Stagno d’Alcontres 31, I-98166 Messina, Italy; carmelo.corsaro@unime.it (C.C.); gabriele.orlando1@studenti.unime.it (G.O.); gabriele.costa@studenti.unime.it (G.C.); mariangela.latino@cnr.it (M.L.); mezzasalma@unime.it (A.M.M.); fneri@unime.it (F.N.); 2 CNR-Institute for Chemical and Physical Processes (IPCF), Viale F. Stagno d’Alcontres 37, I-98158 Messina, Italy

**Keywords:** surface texturing, surface wettability, laser-induced periodic surface structures, metal surfaces, air humidity

## Abstract

The laser surface texturing (LST) technique has recently been used to enhance adhesion bond strength in various coating applications and to create structures with controlled hydrophobic or superhydrophobic surfaces. The texturing processing parameters can be adjusted to tune the surface’s polarity, thereby controlling the ratio between the polar and dispersed components of the surface free energy and determining its hydrophobic character. The aim of this work is to systematically select appropriate laser and scan head parameters for high-quality surface topography of metal-based materials. A correlation between texturing parameters and wetting properties was made in view of several technological applications, i.e., for the proper growth of conformal layers onto laser-textured metal surfaces. Surface analyses, carried out by scanning electron microscopy and profilometry, reveal the presence of periodic microchannels decorated with laser-induced periodic surface structures (LIPSS) in the direction parallel to the microchannels. The water contact angle varies widely from about 20° to 100°, depending on the treated material (titanium, nickel, etc.). Nowadays, reducing the wettability transition time from hydrophilicity to hydrophobicity, while also changing environmental conditions, remains a challenge. Therefore, the characteristics of environmental dust and its influence on the properties of the picosecond laser-textured surface (e.g., chemical bonding of samples) have been studied while monitoring ambient conditions.

## 1. Introduction

Surface processing with ultra-short laser pulses (USPs) is rapidly establishing thanks to its reliability and to the improved understanding of laser–matter interaction mechanisms. The versatility of laser micromachining (LM) is exploited via an extensive range of workstations optimized in all their components (pulsed laser sources, scanners, optics, positioning axes, etc.). The considerable productivity, high precision, easy operability, and robustness of LM make the technique effective in many different fields. Surface processing by USP is presently used to produce molds for micro-optics, to perform drilling for microfluidic devices in borosilicate glass and polymers, so as to obtain structures designed at a micro-/nanometric scale to control surface properties such as wettability and friction [1,2]. So, laser micromachining may be efficiently used for surface engineering applications in different materials [3].

A specific patterned surface is traditionally formed through abrasive blasting, reactive-ion etching, lithography, and mechanical machining. With respect to such conventional approaches, laser technology allows for generating the most controllable and well-defined geometries; it is an environmentally friendly technique since it does not a need chemical reagent, and it does not produce significant waste [4,5,6]. Laser-textured shape and textured area fraction have a strong influence on the wetting behavior and bacterial adhesion of metallic (Ti–6Al–4V), ceramic (hydroxyapatite), and polymeric (polymethyl methacrylate) surfaces, all of which are relevant for biomedical, energy, and mechanical applications. Surface micro-texturing has been broadly applied to improve tribological properties, such as friction behavior and anti-wear [7]. Laser processing is also used to fabricate micro-/nanostructures, achieving some specified functions, such as colorization and superhydrophobicity. Finally, the effects induced on the textured surface, including chemical modifications, are very interesting since they include extreme wettability and durability conditions [8,9]. Despite these advantages, the laser surface texturing (LST) field is still open to the development of specific process strategies based on the application and the unique features of the laser source, from beam shaping to speed and scan geometries, using a single beam or multiple beams [10]. Among the effects induced by laser machining, the modification of surface wettability, especially in terms of water adhesion on the textured surface, is one of the most important for different applications [11,12,13].

One experimental determination of surface wettability can be achieved by measuring the so-called water contact angle (θw), being the angle at the three-phase line of a water drop in equilibrium on a solid surface; hence, it corresponds to the angle formed between the intersection of the tangent at the solid–liquid interfaces. Solid surfaces showing θw > 90° are hydrophobic because water does not adhere to them, while surfaces with θw < 90° are hydrophilic [14]. Air exposure over time can modify the wetting behavior of laser-textured surfaces [15,16,17,18,19]. The friction and wear resistance parameters are strongly influenced by the texture density and shape (size and depth) imprinted on the engineering materials and vary in dry or lubricating conditions [20,21].

Recently, laser texturing has also contributed to solving a significant problem related to the use of coatings, which possess several undesirable features: for instance, they can change the color of the solar panel, contain toxic materials, and need to be reapplied every year. Without applying any coating, laser surface functionalization by itself has endowed panel materials with the ability to repel water and oil (hydrophobicity and oleophobicity) [22,23]. Laser surface texturing is a relatively well-known technique used to enhance the surface topography of engineering materials; it is specifically used to enhance adhesion bond strength in surface coatings, and improve lubrication for surface contact and the fabrication of superhydrophobic surfaces [3]. Ultra-short laser treatments allow for controlling the degree of wettability from superhydrophobic, which means highly water-repellent, to superhydrophilic. This capability could enable the fabrication of new medical devices, microfluidics, sensors, and other precision parts, and devices with controlled and patterned surface properties [24,25].

In this paper, we describe the results obtained in our laboratories by performing laser micromachining on four different metallic materials using a picosecond laser source. The possibility of tuning the contact angle by adjusting the surface texture is discussed, analyzing the impact of the wettability on the formation of nucleation sites close to the micro-/nanostructured solid/fluid interface, taking into account the increase in the specific surface area of interaction through the structuration of the solid substrate.

## 2. Materials and Methods

### 2.1. Materials

Molybdenum and titanium foils with thicknesses of 0.3 and 0.5 mm and purity values of 99.95 and 99.6%, respectively, were bought from MaTecK (Juelich, Germany). Nickel and silver foils with a thickness of 0.25 mm and purity values of 99.98 and 99.95%, respectively, were bought from Goodfellow. We repeated the texturing process and the corresponding analyses three times per material, using a different foil each time.

### 2.2. Laser Micromachining by an Ultrashort Picosecond Laser Source

The laser source available to the micromachining system is of the “mode-locked” type, offering the capability to modify the frequency of the pulse train (each 15 ps wide) up to 1 MHz, including their number and separation up to 20 ns. These parameters are essential for optimizing the quality of the so-called “cold” ablation process, which guarantees “clean” processing on the 5–50 micron scale. For our purpose, we employed a picosecond laser (532 nm), with 160 kHz as the repetition rate, and a pulse width of 6–8 ps. The laser spot, about 25 μm in diameter, was directed onto the surfaces of different foils using a galvanometric scanner with a telecentric objective (163 mm focal length).

**Figure 1:**
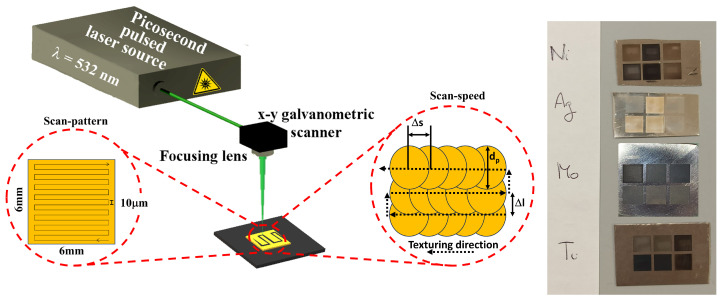
Scheme of the micromachining setup equipped with a picosecond pulsed laser source, an x-y galvanometric scanner. The material’s surface design drawn by the picosecond laser beam (left enlargement). The right enlargement is a representation of the spots overlap during texturing, with Δl being the inter-path distance (defining the vertical spots’ superposition), $d_{p}$ is the spot diameter, and Δs is the distance between two subsequent spots, which is indeed determined by the scanning speed. The panel on the extreme right shows the pictures of samples after texturing processes.

Figure 1 displays the experimental setup, a representative scheme of the chosen texturing geometry, and the pictures of all the samples after the texturing processes. Table 1 summarizes the picosecond laser source specifications and the selected texturing parameters used to treat the investigated samples.

The laser power was fixed at about 1 W. We realized parallel groove patterns, each about 16 µm wide and covering a 6 × 6 mm^2^ area, by running a single mark loop and changing the scan speed from 10 to 2500 mm/s. To obtain an effective overlapping region between machined grooves, the interline separation was set to about 10 µm. The final goal was to achieve uniform machining over a selected area to obtain a specific degree of roughness. After micromachining, all samples underwent ultrasonic treatment in water to remove unwanted residues, thus ensuring the clean, textured surface contributed to reduced exposure to air particulates. Since the goal of this work is to achieve high-quality surface topography of metal-based materials for the proper growth of conformal layers, we explore the correlation between the morphological changes induced by the laser and the wettability properties. To achieve this purpose, after changing different parameters, such as repetition rate and laser power, we decided to keep the last two parameters fixed at 160 kHz and 1 W, respectively, and to only alter the scan speed values, since it is known that, upon increasing the scan speed, the laser residence time decreases. Thanks to the high cooling rates, the laser treatment allows for the attainment of very small grain sizes. Otherwise, if the residence time is sufficient, the thermal cycle may favor grain coarsening. So, the micromachining parameters should be optimized to minimize undesirable effects. Thus, we have tuned the laser/scanner heat parameters to make both hard (micromachining treatment with significant material removal) and soft surface changes (texturing processes aimed solely at modifying wettability and/or removing the oxide layer due to air contamination). This approach induces not only morphological changes but, in some cases, also surface chemical and wettability modifications, all of which affect several technological applications.

### 2.3. Samples Topography and Morphological Analyses

A Tencor Alpha-Step^®^ 500 stylus profilometer (KLA—Milpitas, CA, USA), with a nominal vertical resolution of 0.1 nm, was used to measure step height and surface roughness (defined as the root mean square of the height deviations averaged along the scan length, Rq) of the investigated samples. An X-Y stage enables the mapping of the 2D step height and surface roughness measurements. We used a stylus scan speed of 200 μm/s to obtain the whole profile, and 10 μm/s for the lateral profile and for evaluating the surface roughness. Scanning electron microscopy (SEM) images were recorded using a Zeiss (Oberkochen, Germany) Merlin^®^ FE-SEM equipped with a GEMINI II electromagnetic/electrostatic objective lens system, operating at 20 kV.

### 2.4. Contact Angle Analyses

**Figure 2:**
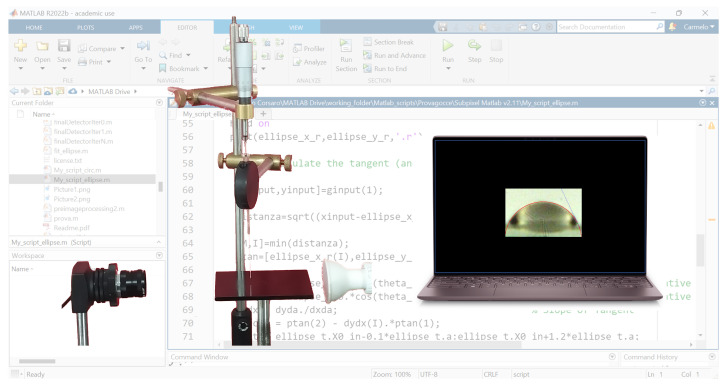
The customized contact angle setup.

A customized setup, shown in Figure 2, was used to perform contact angle (CA) measurements to determine the surface wettability. CA measurements were carried out in time by preserving the sample in a humid air environment. The homemade CA apparatus included a white LED lamp (580 lumens at 6500 K), a UI-146xLE-C uEye camera (½” CMOS sensor), and a microliter syringe driven by a micrometer screw. A 2 μL drop of distilled water was gently released onto the sample placed on the stage, keeping the needle at a distance of less than one centimeter to minimize the effects of gravity on the drop shape. We note that gravitational actions can be neglected for small drop volumes and non-extreme contact angles. Moreover, according to Extrand and Moon [26], gravitational effects can be ignored for a water droplet volume lower than 5 μL on hydrophilic surfaces (with θw > 5°) and 2.7 μL for hydrophobic surfaces (with θw < 160°).

The drop images were captured with a high-definition camera, using an optical lens with a 16 mm focal length and a maximum aperture of 1:1.4 to magnify the picture. Subsequently, the images were analyzed by a custom MATLAB^®^ R2022b (Mathworks, Natick, MA, USA) script to evaluate the contact angle. The MATLAB procedure uses active contours (energy minimization) to track the edge of the drop and fits its profile to a circle or an ellipse. We implemented a MATLAB procedure to quantify the contact angle values, which were measured indirectly by drawing a tangent to the profile at the three-phase contact point after the profile was magnified by photography.

### 2.5. X-ray Photoelectron Spectroscopy Analyses

X-ray photoelectron spectroscopy (XPS) spectra were collected by using the K-alpha system of Thermo Scientific (Waltham, MA, USA). The instrumentation was equipped with a monochromatic Al-Kα source (1486.6 eV), operating in constant analyzer energy (CAE) mode. The pass energy was 20 eV for high-resolution spectra with a spot size of 400 μm. Automated source optimization and gas handling ensured excellent performance and reproducibility. Further, the K-Alpha XPS setup allowed us to go beyond the surface with an ion source (ion energy 2000 eV, etch time 60 s) in order to remove contamination and have a clean surface sample.

## 3. Results

### 3.1. Profilometric Data and SEM Characterization

**Figure 3:**
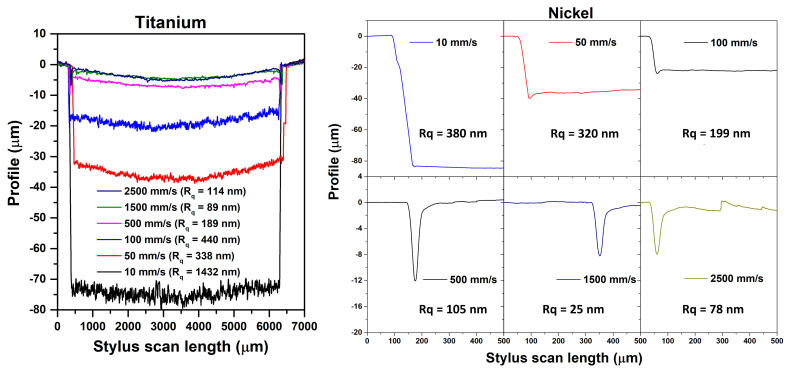
**Left**: channel cross-sectional profiles obtained by profilometry on titanium samples textured using a single mark loop and a scan speed from 10 to 2500 mm/s). **Right**: channel lateral profile obtained by profilometry on nickel samples textured with a single mark loop and a scan speed from 10 to 2500 mm/s).

The left panel of Figure 3 shows the channel’s cross-sectional profiles obtained by profilometry on titanium samples that were textured (6 × 6 mm^2^) using a single mark loop and a scan speed ranging from 10 to 2500 mm/s. The cross-section profiles show a rather uniform width but vary in depth, showing a U-shaped bottom profile in contrast to the V-shaped profile typically achieved through direct laser ablation, for which a certain number of consecutive laser pulses, N, are focused on the same spot. The groove depth (Rz) is the highest (about 80 μm) at the lowest scan speed (10 mm/s); upon increasing the scan speed (from 50 to 500 mm/s), the groove depth is reduced from 38 μm to 7 μm; at the highest speed (1500–2500 mm/s), the groove depth does not exceed 5 μm.

As the root mean square roughness (Rq) is one of the most used parameters for describing the surface roughness at the microscopic level [27], in Table 2, we estimated and reported the values of Rq together with those of Ra (arithmetic average of the profile heights over the considered length) and Rz (maximum height of the profile, excluding the edges) for all the investigated samples and texturing conditions. For titanium, the root mean square roughness is one order of magnitude higher for the lowest scan speed (Rq = 1432 nm) with respect to that observed at the highest speed value (Rq = 114 nm at 2500 mm/s). Moreover, the tendency toward a concave planar bottom surface progressively increases with the scan speed. Similar trends are shown by Ra parameters as a function of scan speed.

The right panel of Figure 3 reports lateral profiles (i.e., at one of the edges) of nickel samples treated with the same micromachining parameters. For scan speeds from 10 mm/s to 100 mm/s, we achieved a well-defined groove pattern with a max channel depth (Rz) of about 80 μm; on the other hand, for scan speeds higher than 500 mm/s, the groove depth was almost null, apart from a point at the edge region (also textured vertically in the passage from one line spacing to another; see Figure 1). This behavior could be ascribed to laser remelting effects, which mainly depend on material composition, hardness, and friction degree [28,29,30]. Overall, as expected, the Rz value decreases upon increasing the scan speed for all the investigated samples.

**Figure 4:**
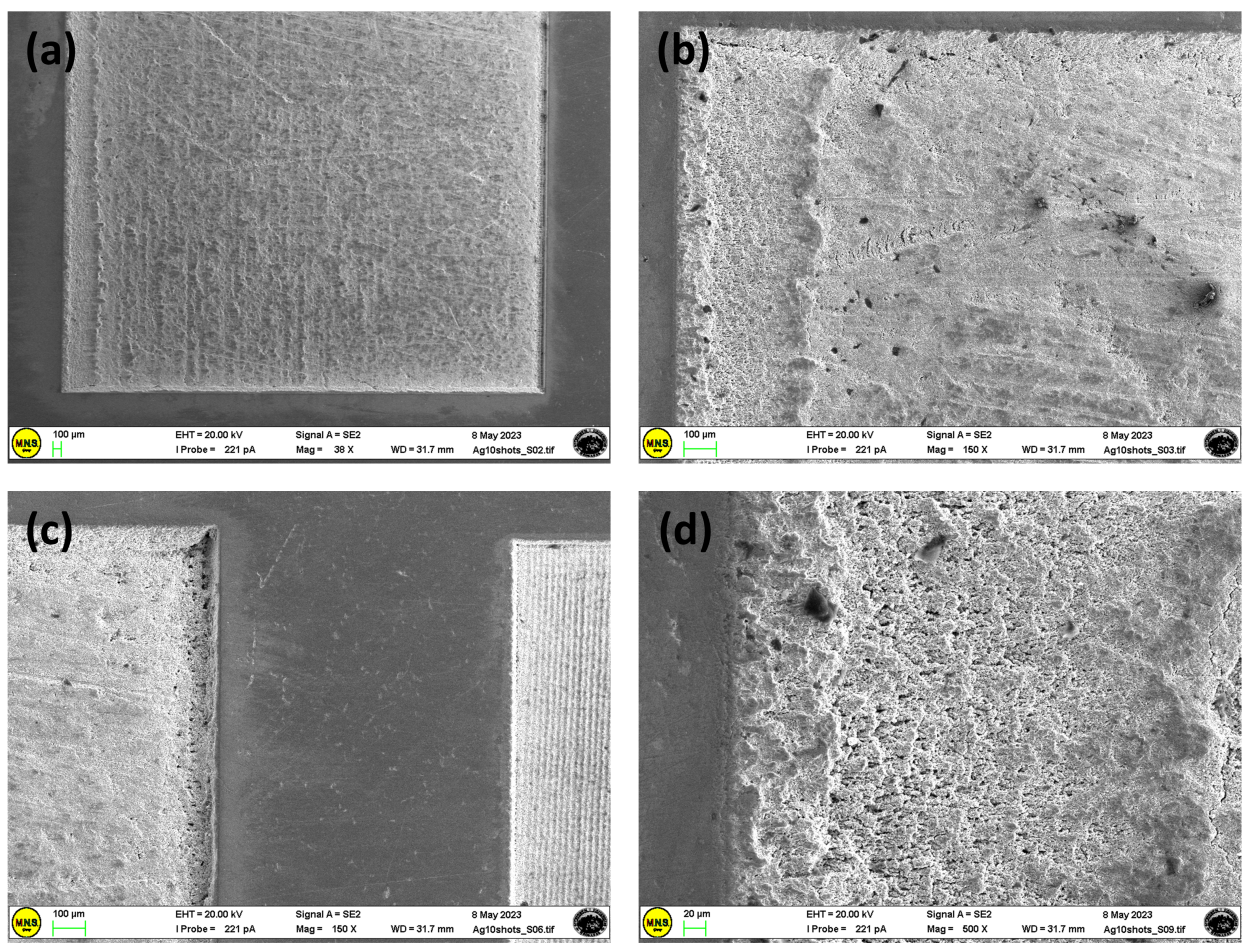
SEM images of the Ag sample textured at a scan speed of 10 mm/s, at different magnifications and points (**a**–**d**).

As shown in Figure 4, for the Ag substrate, a small number of laser scans (10 mm/s) produces poorly defined grooves and microholes on the sample surface. At the lowest scanning speeds, the inside of the molten pool can flow to both sides, allowing mass and heat transfer with the formation of a dense modified layer [31]. The high local energy accumulation on the sample surface favors the formation of material debris in the remelting layer (Figure 4), causing the material to vaporize by destroying the formed layer. Furthermore, the internal thermal stress provoked by a large temperature gradient on cooling allows for the formation of microcracks in the remelted layer.

**Figure 5:**
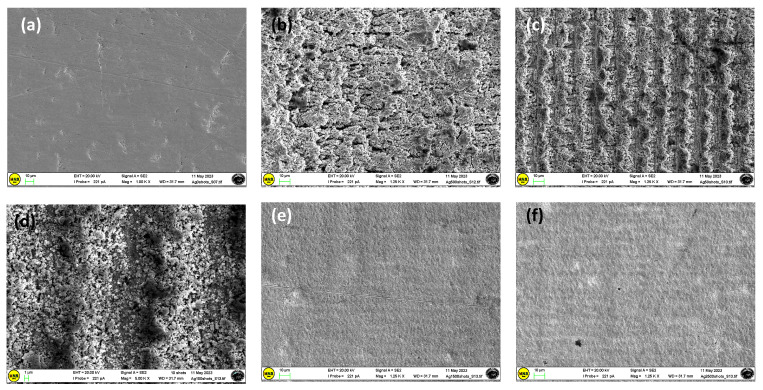
Comparison among the texturing processes at different scan speeds for the Ag sample. (**a**) untextured; (**b**) textured at 10 mm/s; (**c**) 50 mm/s; (**d**) 100 mm/s (**e**) 1500 mm/s; (**f**) 2500 mm/s.

In addition, as shown in Figure 5, textured Ag surfaces are very different after ps-laser texturing at different scan speeds. At the lowest scan speed (10 mm/s), remelting areas are produced (Figure 5b), and upon increasing the scan speed at values of 50 and 100 mm/s (Figure 5c,d), specimens are mainly composed of columnar grains (with many submicron second-phase particles (SPPs) inside them), which are coarser than the grains in the substrate.

**Figure 6:**
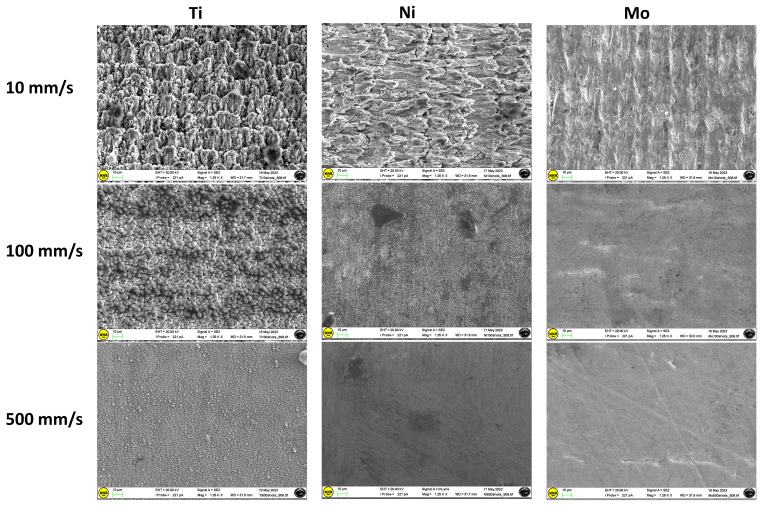
Representative SEM images of titanium, nickel, and molybdenum sheets textured at different scan speeds: 10 mm/s, 100 mm/s, 500 mm/s.

**Figure 7:**
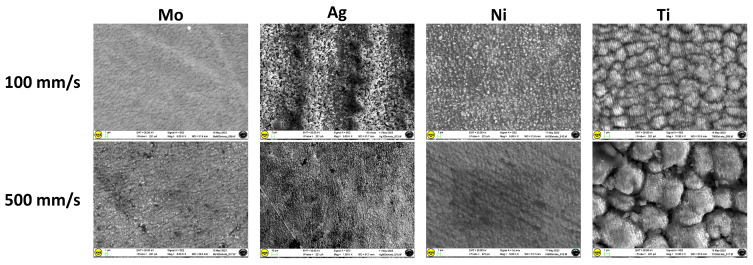
High-resolution SEM images of the samples textured at two different scan speeds.

The concentration of nano-/microstructures over the structured area increases as the scan speed decreases. The decrease in scan speed causes an increase in the number of laser pulses per unit length along the scanning line. This results in more overlapping of the laser pulse, determining more deepening of the crater formed by the last pulse by the upcoming pulse. Further, the decrease in scan speed results in an increase in the width of the groove. At higher scan speeds (1500 and 2500 mm/s), the Ag surface morphology remains almost unchanged throughout the process with respect to the untextured one (Figure 5e,f compared with Figure 5a). The obtained groove of 6 × 6 mm^2^ is well defined for all of the studied samples, especially at the lowest scan speeds. Representative SEM images of titanium, nickel, and molybdenum sheets textured at different scan speeds (10 mm/s, 100 mm/s, and 500 mm/s) are presented in Figure 6, and more morphological details emerge from higher-resolution SEM images (Figure 7).

The protrusions on the surface of the material begin to form according to the morphology of “fish scales”, which is mainly evident for the titanium target. Ni and Ti targets processed at a rather fast scan speed (500 mm/s) show evident differences in surface morphologies: the Ni target presents an almost uniform surface with long-extending regular “parallel grooves”; conversely, the similarly treated Ti target reveals packed “ringlike” structures, which are presumably markers of a resolidified melt. The “parallel-grooves” pattern is still present inside the structures, with similar dimensions and periodicity to those observed for Ni.

### 3.2. Contact Angle Data

**Figure 8:**
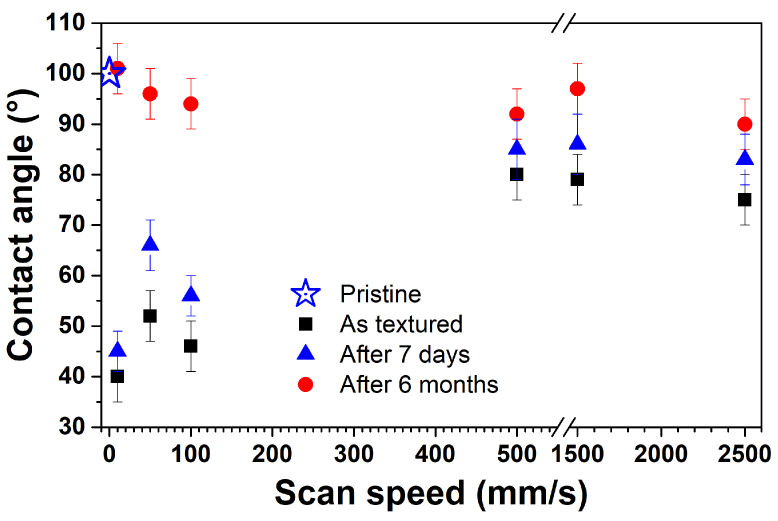
Contact angle values for the Ti sample immediately after texturing and six months post-texturing as a function of the laser micromachining scan speed.

The relationships between the material’s surface wettability and morphology changes were studied by carrying out contact angle (CA) measurements. First, we examined the surface wettability of titanium surfaces over time since the effects of hydrophilicity/hydrophobicity on titanium sheets are uncertain and poorly understood. However, it is largely known that the age of the titanium sample affected its wettability. This is significantly influenced by contaminants (i.e., hydrocarbon pellicle) accumulated on its surface or by the titanium surface composition change over time [32]. Figure 8 shows CA images of both untextured and textured titanium sheets, immediately after laser texturing and again after 1 week and 6 months. The samples underwent a simple ultrasound cleaning process, commonly used in many industries, to eliminate air contamination before CA measurements.

Looking at these data and their designed morphologies (evidenced by SEM images), we conclude that the hydrophobic character of titanium has been controlled by appropriately modifying the shapes and sizes of the textured patterns. CA values range from 40° to 80° depending on the scan speed. We observe that after 1 week, and for scan speed values up to 100 mm/s, CA values were around 50° again, while complete surface hydrophobicity was restored after 6 months of ambient air storage. Hence, in this case, it emerges that CAs increase in time, regardless of the morphological pattern obtained, and remain almost constant after 6 months, reaching a saturation value in the 90–100 range. Otherwise, as the scan speed increases to 500 mm/s, the CA trend is not different for as-textured and aged samples (starting from a week). Based on this evidence, we conclude that the laser-induced morphological changes affect the wettability transition only under specific conditions. These changes could be ascribed to the changes in the Ti surface chemical composition, leading to its hydrophobic character [32]. In the next section, XPS data are discussed in order to discern this aspect.

**Figure 9:**
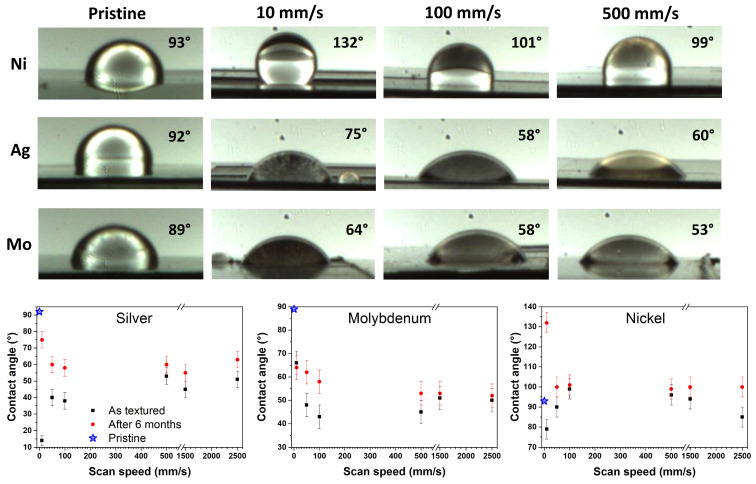
Example of contact angle images for Ag, Mo, and Ni samples, pristine and textured at different scan speeds: 10, 100, and 500 mm/s. Contact angle trends for the considered textured samples as functions of the scan speed; measured immediately after texturing and 6 months later.

The same CA analyses were carried out on the other investigated materials. Figure 9 reports the contact angle trend for Ag, Ni, and Mo samples as a function of scan speeds for as-textured and aged conditions (data after 6 months are plotted, as they are the same as those collected after 1 week). All the investigated samples exhibited a trend from mostly hydrophilic to almost hydrophobic. In particular, under all texturing conditions, Ni showed a more hydrophobic character, while Ag and Mo remained essentially hydrophilic.

Note that the Ni sample, after 6 months of texturing, shows hydrophobic properties at 10 mm/s, even exceeding the pristine material. At the lowest scan speed, the laser surface remelting has a remarkable influence on Ni hardness, which in turn affects its hydrophobicity, as known from the literature [4]. In fact, the laser’s thermal effects primarily involve both the near-surface softening layer and the subsurface metamorphic layer. These effects are more pronounced for the Ni sheet (both pristine and treated), and are less hard than the other investigated materials. Nevertheless, the results demonstrate that it is possible to construct hydrophobic surfaces on hydrophilic substrates by laser tailoring the surface microstructure, without the additional use of low surface energy materials.

**Figure 10:**
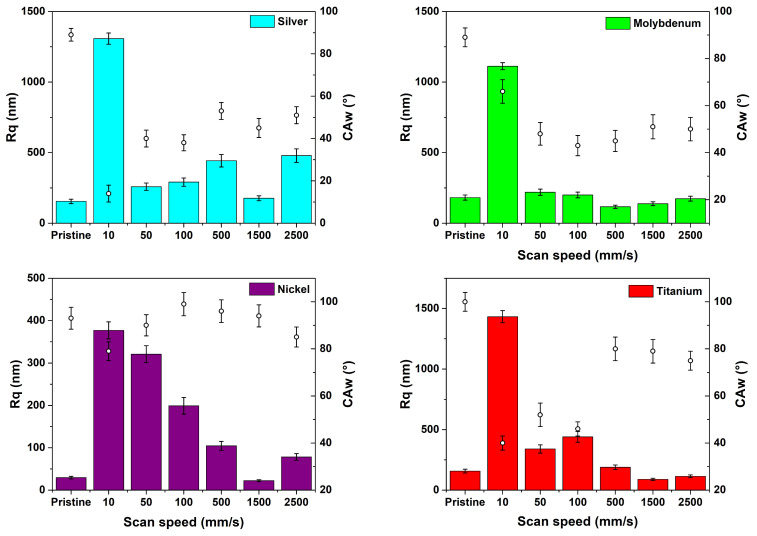
Roughness (left-y bar plot) and water contact angle (right-y scatter plot) measured for the as-textured samples as functions of the texturing scan speed.

Overall, specific laser texturing processing conditions allow for the tuning of CA and average roughness; this is evident when looking at trends of CA and roughness values versus the scan speed (see the double-y plot of Figure 10).

We outline that the changes obtained using our picosecond-texturing protocol are comparable to those found by using femtosecond pulses [33]. However, texturing-induced morphological changes do not always lead to the same CA variations for each of the studied materials. This provides further evidence that the laser-induced modification of surface wettability cannot be correlated solely with surface morphology.

## 4. Discussion

The picosecond laser-textured surfaces show different types of morphology by changing the pulse energy, E, calculated as E=P/v, where P and v denote the laser power and the scanning speed, respectively. Decreasing the scanning speed has an effect on the evolution of surface morphologies similar to that of increasing the laser fluence [34,35,36].

By reducing the scanning speed, the total energy per area deposited onto the surface can increase. In this case, the spot overlap rate is higher and the material absorbs more laser energy, with a stronger ablation effect. During micromachining, laser spots will overlap each other on the sample surface; their overlap depends on the texturing scan speed, as schematized in Figure 1. The spot overlap ratio is obtained by the following relation:

(1) $$\psi =\frac{d-v/f}{d}=1-\frac{v}{d\cdot f}$$

where ψ is the spot repetition rate; d is the spot diameter; v is the scanning speed; f is the laser repetition frequency; and v/f is the spot center spacing [31]. Table 3 reports the values of the spot overlap ratio as a function of the texturing parameters.

According to the above relation, the distance between adjacent spots increases with the scanning speed, contrary to their overlap, the number of pulses per unit area and, thus, the laser energy deposited onto the sample surface. Hence, by varying the scanning speed values, the formation of different microstructures on the surface of the material can be induced. At low values (i.e., 10 mm/s), a higher ablation rate is obtained, leaving a rather rough surface morphology (on a micrometer scale), yet without producing any microhole. A clear separation between protrusions and depressions cannot be seen and the bulge distribution is irregular. Laser ablation dominates the treatment. The effect of a low scan speed on the evolution of the surface morphologies is similar to that generally observed by increasing laser fluence. These aspects are peculiar in the choice of appropriate working conditions to tune the chemical–physical properties of the materials of interest. We observed that, for Mo and Ag substrates textured at scan speeds of 50, 100, and 500 mm/s, the roughness of these samples is relatively low, and the line-patterned microstructures are similar despite being the most hydrophilic ones. In addition, since the surface roughness does not depend on time, the corresponding change in wettability must correspond to variations in the surface chemistry.

**Figure 11:**
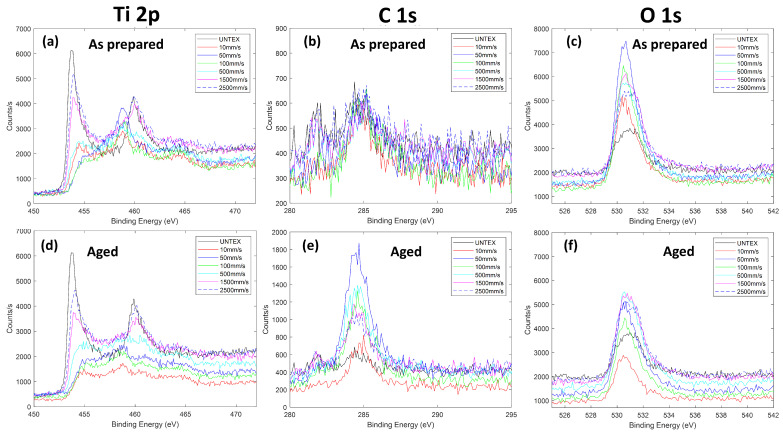
High-resolution XPS profiles of titanium samples untextured and textured at different scan speeds just after texturing (“as prepared samples”) and aged for six months; (**a**–**c**) panels, respectively, refer to the Ti 2p, C 1s, and O 1s profiles for as-prepared samples, whereas (**d**–**f**) panels refer to the same elemental profiles for the aged samples.

In order to study the laser-induced effects on the surface chemical composition and bonding configurations, as well as the in-time evolution of the surface wettability, XPS measurements were carried out. Here, we discuss the behavior observed for the titanium samples before and after texturing (until about 30 days of ambient air storage). Figure 11 and Table 4 show high-resolution XPS Ti 2p, C 1s, and O 1s spectra, as well as the chemical composition estimated by analyzing the XPS survey spectra. First, we observe that two types of Ti 2p spectra characterize the samples by varying the scan speed.

In particular, we observed the same Ti 2p profiles for the untextured samples and those textured with scan speeds of 1500 mm/s and 2500 mm/s. These profiles are mainly dominated by the metallic Ti peak centered at about 453.9 eV. Otherwise, the Ti samples textured by varying the scan speed from 10 mm/s up to 500 mm/s are mainly characterized by the titanium oxide features expected at around 455.5 eV (Ti II), 457.3 eV (Ti III), and 458.7 eV (Ti IV). The same XPS profiles occurred in the aged samples. However, for these samples, regardless of the laser processing, the carbon content increases while the oxygen content decreases (Table 4). We show that, before XPS acquisitions, an ion etching process was carried out to remove adventitious carbon species for all the investigated samples. In addition, XPS data indicate the presence of a low nitrogen percentage (less than 3%), due to air contamination, potentially leading to Ti-N bonds; their contribution is located at around 455 eV, overlapping with the Ti(II) oxide band [37].

The in-time accumulation of carbon species, favored by the porosity of the textured surfaces, may induce the generation of new hydrophobic functional groups, such as -CH_3_, which are generally hydrophobic. In addition, the observed changes could also be due to the decomposition of carbon dioxide in air during laser processing [15] as well as to a large amount of polar oxide and hydroxide sites accumulated on the treated surface. Finally, the adsorption of airborne organic constituents with polar functional ends onto the active polar sites of the textured surface cannot be excluded. All these mechanisms could have occurred in our samples, potentially affecting the material’s wetting state, which appears to be unstable over time (30 days).

## 5. Conclusions

Visible picosecond laser enabled single-step fabrication of microstructures on different metallic materials. Spatially tuned micromachining allowed full surface coverage of redeposited micro-/nanoaggregates. Periodic parallel lines are often combined with microporous structures (nearly regular ripples), which, for some of the textured materials, reduced separation and improved spatial uniformity. Tunable water-wetting behavior was observed, depending on the morphological features of the samples, which vary significantly as a function of the scan speed. The in-time modification of surface wettability is instead due to the change in surface chemistry. XPS data prove the accumulation of carbon and the generation of new hydrophobic functional groups, which play a critical role in the improvement of surface hydrophobicity. The evidence is interesting but some additional analyses are necessary to explain the final wetting scenario for the investigated metal-based materials. Specifically, understanding the appropriate relationship between the surface roughness, dimension, and distribution of multiscale structures, and surface chemical properties, including dispersive and non-dispersive components, still requires further investigation. In fact, this latter concept is mainly useful for technological applications involving the use of textured polymer-coated metals.

## Figures and Tables

**Table 1:** Specification of the laser system and parameters used for the texturing process.

**Main characteristics of the employed diode-pumped Nd:YVO_4_ laser system**	
**Laser specification**	**Value**
Maximum laser power [W]	10
Wavelength [nm]	532
Pulse width [ps]	6–8
Repetition rate [kHz]	10–1000
Maximum pulse energy [μJ]	60
Laser beam diameter [µm]	23
Focusing length of lens [mm]	163
Maximum scanning speed [mm/s]	3000
Atmosphere	Air
**Chosen laser texturing conditions**	
**Employed variable**	**Value**
Laser power [W]	1
Repetition rate [kHz]	160
Scanning speed [mm/s]	10, 50, 100, 500, 1500, 2500

**Table 2:** Roughness values and maximum profile height in microns for the investigated samples textured at the different scan speeds.

	Nickel			Titanium			Silver			Molybdenum		
	Rq	Ra	Rz	Rq	Ra	Rz	Rq	Ra	Rz	Rq	Ra	Rz
UNTEX	0.03	0.02	–	0.16	0.14	–	0.15	0.11	–	0.18	0.15	–
10 mm/s	0.38	0.32	83	1.43	1.07	75	1.31	1.11	95	1.11	1.01	79
50 mm/s	0.32	0.25	39	0.34	0.27	38	0.26	0.19	71	0.22	0.17	39
100 mm/s	0.20	0.15	22	0.44	0.35	21	0.29	0.23	35	0.20	0.15	22
500 mm/s	0.10	0.07	2	0.19	0.15	7	0.44	0.36	31	0.12	0.08	8
1500 mm/s	0.02	0.01	1	0.09	0.05	5	0.18	0.14	2	0.14	0.09	6
2500 mm/s	0.08	0.05	1	0.11	0.09	5	0.48	0.38	1	0.17	0.15	3

**Table 3:** Some texturing parameters (d = laser spot size; f = laser repetition rate; v = scan speed) and the estimated laser spot overlap ratio.

d (μm)	f (kHz)	v (mm/s)	ψ
23	160	10	0.997
23	160	50	0.986
23	160	100	0.973
23	160	500	0.864
23	160	1500	0.592
23	160	2500	0.320

**Table 4:** Elemental composition in % from XPS analyses of titanium samples untextured and textured at different scan speeds just after texturing (“as prepared”) and aged for six months.

	As Prepared			Aged		
	C	Ti	O	C	Ti	O
UNTEX	25.96	36.86	37.17	25.96	36.86	37.17
10 mm/s	21.58	28.44	49.98	36.40	23.88	39.72
50 mm/s	11.55	38.86	49.59	24.83	47.75	27.42
100 mm/s	23.12	24.29	52.59	35.29	24.17	40.54
500 mm/s	26.86	27.89	45.25	32.32	22.91	44.77
1500 mm/s	20.67	28.59	50.74	28.31	31.73	39.96
2500 mm/s	27.45	22.28	50.27	24.18	37.66	38.16

## Data Availability

The data presented in this study are available on request from the corresponding author. The data are not publicly available due to ethical reasons.

## Abbreviations

The following abbreviations are used in this manuscript:

LST	laser surface texturing
USP	ultra-short laser pulses
CA	contact angle
SEM	scanning electron microscopy
XPS	X-ray photoelectron spectroscopy
